# The effect of redox signaling on extracellular matrix changes in diabetic wounds leading to amputation

**DOI:** 10.1016/j.bbrep.2021.101025

**Published:** 2021-05-21

**Authors:** Mohamad Hadi Saeed Modaghegh, Shirin Saberianpour, Sakineh Amoueian, Jamal Jalili Shahri, Hamidreza Rahimi

**Affiliations:** a Vascular and Endovascular Surgery Research Center, Mashhad University of Medical Sciences, Mashhad, Iran; b Departement of Pathology, Emam Reza Hospital, Mashhad University of Medical Sciences, Mashhad, Iran; c Departement of Medical Genetics and Molecular Medicine, Mashhad University of Medical Sciences, Mashhad, Iran

**Keywords:** Diabete, Redox, wound, signaling, ECM, extracellular matrix, MDA, Malondialdehyde, SOD, Superoxide dismutase, NO, Nitric oxide, ELISA, The enzyme-linked immunosorbent assay, ROS, Reactive oxygen species, MMP, Matrix metalloproteinase, HIF, Hypoxia-inducible factor

## Abstract

**Introduction:**

& Objectives: Redox signaling is a critical regulator in the process of wound healing. This signaling pathway can be effective in the development or healing of diabetic ulcers through the ECM.In this study, the structure of extracellular matrix investigated in relation to redox signaling in the tissue of patients with diabetic ulcers that lead to organ amputation.

**Materials and methods:**

The case-control design on diabetic patients ulcers as case group and non-diabetic limb ischemia as control were used.Hematoxylin-eosin, trichrome, and elastin staining methods were used for pathological evaluations of ECM. MDA, total thiol, and SOD levels were measured using ELISA kits to assess the oxidative stress level. Also, NO level was measured by using ELISA kits in both groups. Expression levels of genes MMP2, MMP9, and HIF were detected using real-time PCR with SYBR-green assay.

**Results:**

The pathological results showed an increase in the thickness of collagen and elastin fibers. Lipids atrophy was visible in the tissue isolated from the diabetic wound group. The amount of MAD to evaluate the level of lipid oxidation in patients with diabetic Ulcer was significantly higher than the control group(p < 0.01). Thiol level was significantly lower in the diabetic ulcer group than in the control group(p < 0.0001). The expression of metalloproteinases 2 and 9 genes in the tissues isolated from diabetic ulcers was lower than the control group(p < 0.0001). While the expression of the HIF gene in this group was higher than the control group(p < 0.0001).

**Conclution:**

In the diabetic wound, the HIF secretion due to hypoxic conditions is beneficial for matrix deposition and prevents protease activity, but if the hypoxia persists, it can lead to ECM deposition subsequently increases the tissue pressure, increases of the collagen I-to-collagen III ratio in collagen accumulation that due to more hypoxia , lipidsAtrophy and eventually amputation.

## Highlights

❖ Lipid oxidation initiates the pathway of adaptation to hypoxia and production of HIF factor in diabetic wounds
❖ In the diabetic wound, the HIF secretion due to hypoxic conditions is beneficial for matrix deposition and prevents protease activity.
❖ Persistence of hypoxia in diabetic wound can lead to ECM deposition
❖ ECM deposition subsequently increases the tissue pressure, increases of the collagen I-to-collagen III ratio in collagen accumulation that due to more hypoxia, lipids Atrophy and eventually amputation

## 1 Introduction

The term “diabetic foot” defines a wide range of abnormalities, including the foot prone to ulceration due to diabetes (neuropathic foot and the foot with the peripheral vascular disease) and the ulcerated foot [1]. The diabetic ulcer is one of the most important diabetes complications, the life-long risk of which reaches 15% for a diabetic patient [2]. Annually, more than one million diabetic patients undergo leg amputation due to the diabetic ulcer [3]. The amputation prevalence is 15 times higher in diabetic patients than non-diabetic individuals, with the annual mortality of diabetic patients undergoing limb amputation being 39%–68% [4]. Molecules of the extracellular matrix are the essential components of wound healing. First, a temporary extracellular matrix is formed. During tissue repair, the structural integrity of ECM expands and eventually leads to wound healing [5]. Second, ECM molecules regulate cellular function, mediate cellular interaction, and act as a reservoir and functional modulator for cytokines and growth factors [6]. Therefore, a basic evaluation of the cellular structure and signaling involved in the ECM of diabetic ulcers can be a therapeutic strategy for wound healing [7]. Redox signaling is a critical regulator in wound healing. This signaling pathway exerts its effects, particularly through the ECM [8].

Due to increased oxidative stress, redox signaling is widely involved in the development of diabetic complications [9]. The ECM structure and function are impaired in diabetes by fibroblast dysfunction and protein deposition alteration, leading to abnormal ECM structure and composition [10]. The deposition of collagen, the most abundant ECM protein in normal tissue and healing wound, changes significantly in diabetes [11]. One of these changes is the decreased collagen I-to-collagen III ratio, which is associated with a decreased tensile strength of ECM. Also, there can be an increased distance between collagen fibers in the ECM of diabetic tissue. Diabetes may also affect the collagen fibril diameter; however, it is not yet identified whether it increases or decreases the fibril thickness. One of the factors in the development and improvement of diabetic ulcers is the ROS levels of tissue. When the ROS level is the highest, redox signaling-induced wound repair and tissue regeneration are activated [12]. In fact, the ROS can result in the cell proliferation and damaged tissue regeneration by the angiogenesis stimulation, adhesion molecule increase, and inflammatory factor release [13]. ROS compounds increase angiogenesis directly or through HIF stimulation [14].

However, the ECM structure and the chemicals present at diabetic tissue ulcers that lead to amputation should be further studied because these tissues do not respond to routine treatment for other chronic ulcers, so the signaling and damage mechanisms may have taken a different path in these patients( [15,16]).In this study, the structure of extracellular matrixis investigated in relation to redox signaling in the tissue of patients with diabetic ulcers that lead to organ amputation.

## 2 Material and methods

### 2.1 Specimen collection

The present study is a pilot study with a case-control design on diabetic patients suffering from diabetic foot ulcers resulted in amputation. Non-ischemic tissue specimens from patients with non-diabetic limb ischemia were used as control. 10 specimens were evaluated in each group. An inclusion and exclusion criteria were used for matching and prevention from confounding factors in the study and control groups. The inclusion criteria including; patient group having diabetes and not having another underlying disease (Vasculitis, Cangenital heart disease, hypercoagulation state and COPD), control group not having a specific disease(Diabetes, Vasculitis , Cangenital heart disease, hypercoagulation state and COPD), do not take corticosteroids ,no smoking, male gender, and age ranged from 45 to 65 years old.Following the approval of the Ethics Committee of Mashhad University of Medical Sciences(IR.MUMS.MEDICAL.REC.1399.439), the patients with diabetic foot and limb ischemia gave informed consent before wound sampling. The tissue specimens were obtained from the amputated tissues post-surgery. An aliquot of specimens was placed in a cryovial in liquid nitrogen at -80 °C temperature for molecular tests. A specimen 3 cm in length, width, and height was collected in a pathology container with.

10% formalin and was immediately transferred to the pathology department for pathological evaluations.

### 2.2 Pathology

Specimens from patients were put in 10% formalin and immediately transferred to the pathology department of the Imam Reza Hospital. First, the tissues were placed in the tissue processor (Tissue processor, POOYAN model: MIK 2230, serial 223073, made in Iran), and then the tissue passage was performed. The tissues were infiltrated with liquid paraffin and then formed into blocks. The blocks were cut using a microtome, and the sections were mounted on glass slides. Ready-to-stain slides were stained using collagen-specific and elastin-specific staining methods. For elastin-specific staining, the slides were deparaffinized, then immersed in hematoxylin and Weigert's iodine solution for 15 min, and then were rinsed using distilled water. Then they were immersed in Van Gieson's solution for 2–3 min and then dried using a paper. The trichrome staining was used as a collagen-specific staining method. The slides were deparaffinized, then immersed in hematoxylin for 15 min, and then rinsed with distilled water. The slides were immersed in an acid-alcohol solution and then removed immediately. They were then re-washed with distilled water and immersed in phosphomolybdic acid for 5 min and then rinsed again. Aniline Blue Solution was applied on the slides for 5 min and then rinsed with distilled water. A water-acid solution was applied on the slides for 5 min, and then the slides were washed with distilled water, and then were dehydrated. Following mounting the coverslip, the slides were ready for pathological evaluation.

### 2.3 Protein extraction from tissues

200 μL of lysis buffer (NaCl, Tris-HCl, NP-40 supplemented with inhibitor) was added to 10 mg of tissue. The mixture was incubated for 30 min and then homogenized. For removal of the impurities, the mixture was centrifuged at 14000 rpm for 20 min at 4 °C temperature, and the supernatant was isolated. Protein concentration was assessed using the NanoDrop (X).

### 2.4 MDA

The MDA was detected using a Nalondi™ Lipid Peroxidation (MDA) Assay Kit. First, the reagents were left at room temperature for 30 min before the procedure. The reagents R1, R2, and r were mixed in a 1:1:2 ratio for working solution preparation. Then, 200 μL of the protein sample was mixed with 800 μL of the working solution. The tube lids were closed, and the tubes were heated at 95 °C temperature for 45 min using a bain-marie method. Then the samples were placed in ice water for 10 min. The samples were centrifuged at 3000 rpm for 15 min 250 μL of samples of each group were transferred into the wells of a 96-well microplate. The optical density of the supernatant was measured at the wavelength of 550 nm using a spectrophotometer (Bio Tek Instruments, Inc. VT 05404-0998).

### 2.5 SOD

Superoxide dismutase was detected using a Nasdox™ Superoxide Dismutase (SOD) Activity Assay Kit based on the kit protocol. 50 μL of the extracted protein sample from each group (study and control groups) were mixed with 200 μL of Reagent 1, and 50 μL of the Reagent 2 was poured into the 96-well microplate. The incubation was performed for 5 min. The optical density at a wavelength of 405 was measured using a spectrophotometer (Bio Tek Instruments, Inc. VT 05404-0998).

### 2.6 Thiol

The total thiol content was assessed using the Free Thiol Assay Kit (Cat-No: S1180-100, S1180-200). 100 μL of each protein sample was mixed with the dilution buffer to reach 400 μL. Then 100 μL DTNB was added. After 5 min, 100 μL sulfosalicylic acid was added and incubated on ice for 30 min. After 5 min of centrifugation at 12000 rpm, the total supernatant was transferred to a new microtube. 400 μL of the reaction buffer was added to each sample and then was centrifuged at 112000 rpm. Finally, the optical densities were measured at a wavelength of 412 nm.

### 2.7 NO(Nitric oxide)

The NO concentration was assessed using the Natrix™ Nitric Oxide (NO) Assay Kit. Before initiation, all kit solutions were incubated at room temperature for 15–30 min to reach thermal equilibrium. Then 150 μL of each sample was mixed with 80 μL of Buffer A of the kit in a microtube. 80 μL of Buffer B was added, and the mixture was vortexed. The mixture was centrifuged at 14000 rpm for 10 min, and then the supernatant was isolated. 50 μL of the resulted supernatant of each sample was added into the wells, and Reagent R1 was added to each well. The plate was incubated away from light at room temperature for 10 min. Then, 50 μL of Reagent R2 was added to the wells. Following mixing for 10 min, the optical density was detected at a wavelength of 570 nm.

### 2.8 Evaluation of gene expression

The appropriate primers were designed and selected using the Primer3 software (Table 1) and were synthesized by Takapouzist Company to evaluate the expression of metalloproteinase 2, metalloproteinase 9, and HIF genes. Tissue samples stored at -80 °C before the experiments were mechanically homogenized. Then, the total RNA was extracted using the Total RNA extraction Kit, A101231, Pars Tous. The quality of the extracted RNAs was evaluated using a NanoDrop device. cDNA was synthesized based on the extracted RNA concentration using the cDNA Synthesis Kit, A101161, Pars Tous. The expression level of each gene was evaluated using the specific primers and primers of the reference gene GAPDH by SYBRgreen Master Mix and Light Cycle 96 Instrument, REF: 05815916001L. Fold changes were estimated using the ΔΔ Ct method. Statistical analysis was performed using the software GraphPad Prism version 8.4.2 (679).

**Table 1:** Primer lists.

Gene	Sequences(5′-3′)
GAPDH	F:AAGGTGAAGGTCGGAGTCAACG R:TGGTGGTGTGCAGGAGGCATTGC
MMP2	F:CCC ATA CTT TAC TCG GAC CA R:TGA CCT TGA CCA GAA CAC CA
MMP9	F:CCA CCG AGC TAT CCA CTC AT R:GTC CGG TTT CAG CAT GTT TT
HIF	F:CATAAAGTCTGCAACATGGAA R:ATTTGATGGGTGAGGAATGG

## 3 Results

### 3.1 Tissues with diabetic wounds increased elastin and collagen thickness and adipose tissue atrophy

**Fig. 1:**
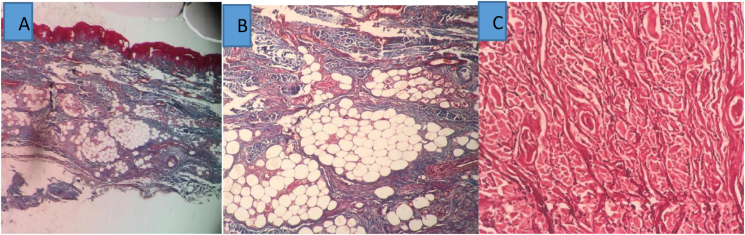
Histopathology Control Tissue: A: No increase in dermis thickness Magnification 4 × . B:Trichrome - no fat atrophy Magnification 10 × C:Elastin - no rupture of elastin fibers Magnification 10 ×

**Fig. 2:**
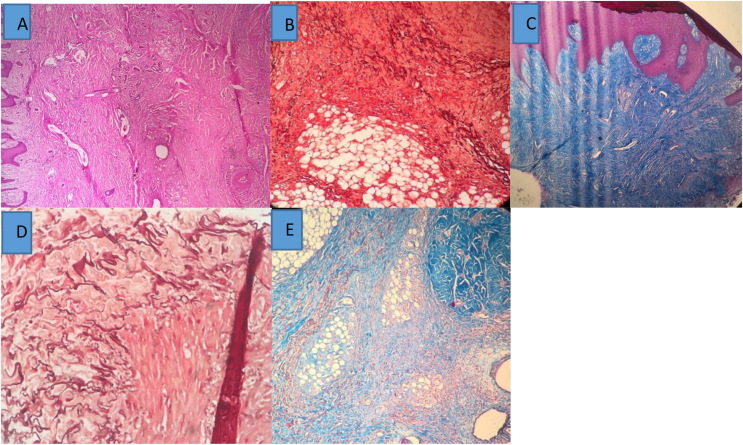
HistopatologyDiabeteTissue.**A**: Hematoxylin eosin-Increases dermis thickness Magnification 4 × **B**:Elastin - Increases elastin fibersMagnification 4 × **C**:Trichrome –Increase Collagen Magnification 10 × **D**:Elastin filament fragmentationMagnification 10 × **E**:Trichrome - fat atrophy Magnification 10×

In pathological examinations, hematoxylin-eosin, trichrome (collagen) and elastin staining was performed. Hematoxylin-eosin staining showed an increase in dermal thickness in the diabetic ulcer group compared to the control group (Fig. 1, Fig. 2). Trichrome staining of diabetic ulcer samples showed increased collagen and increased adipose tissue atrophy in comparison with the control group (Fig. 1, Fig. 2).

### 3.2 Lipid oxidation activity increased in diabetic wound tissues

**Fig. 3:**
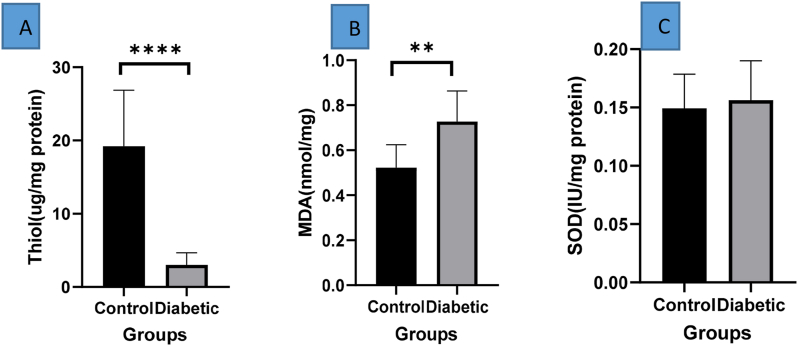
ROS level in Control and Diabetic Group **A**:SOD level did not change in 2 groups **B**:MDA level was increased in diabetic group( **p < 0.01 (n = 3)). **C**:Thiol level was decrease in diabetic group(****p < 0.0001 (n = 3)).

MDA is the final product of lipid peroxidation. In this study, according to the results, it was shown that the rate of lipid peroxidation in the group of tissues containing diabetic ulcers was significantly higher than the control group (**p < 0.01)(Fig. 3).

### 3.3 Superoxide dismutase levels did not change in the diabetic wound tissue group and the control group tissue

Superoxide dismutase (SOD) is one of the key enzymes in the body's antioxidant defense against free radical damage. The results show almost identical levels and no statistically significant difference of superoxide dismutase in the ulcer tissue group. Diabetes and control tissues (Fig. 3).

### 3.4 Proteins oxidation activity reduced in diabetic wound tissue

In this study, it was shown that tissues with diabetic ulcers compared to the control group had a significantly lower difference in oxidation activity on proteins with the release of the thiol group compared to the control group(****p < 0.0001 ) (Fig. 3).

### 3.5 NO levels increasedin the diabetic wound tissue

**Fig. 4:**
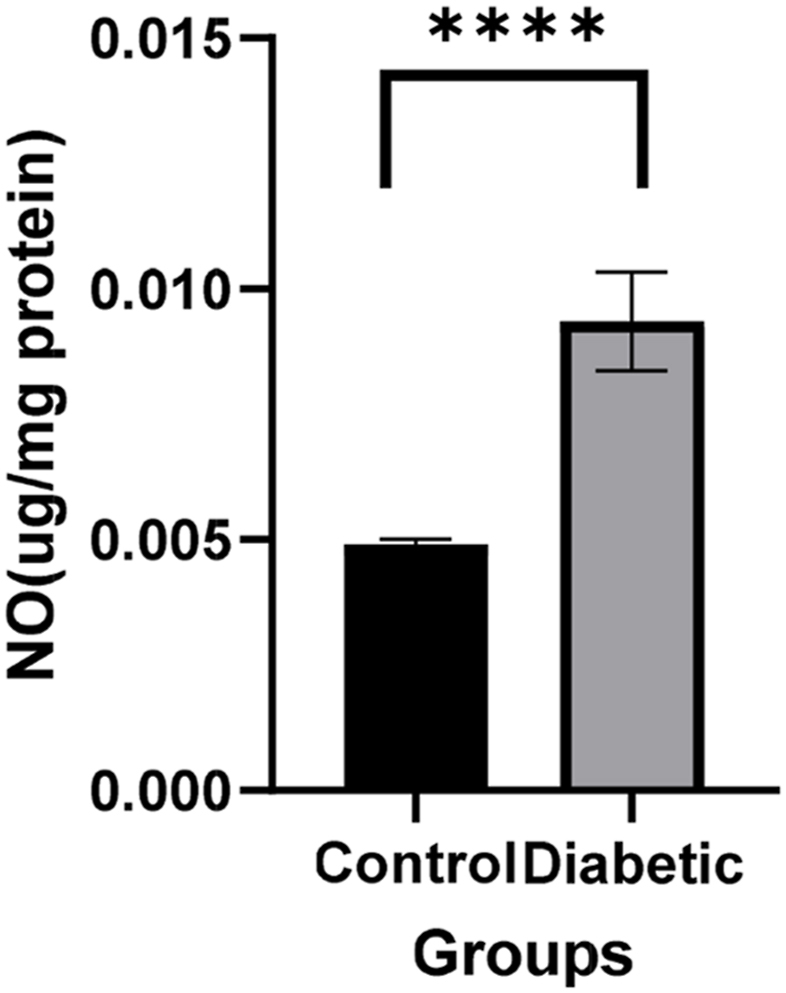
Nitric oxide Level in Control and Diabetic groups. Nitric oxide Level was increased in diabetic group (****p < 0.0001 (n = 3)).

Nitric oxide (NO)is involved in a number of physiological and pathological processes in the body. This compound also has vasodilatory properties in Vessels. The results showed a statistically significant increase in nitric oxide levels in tissue samples with diabetic ulcer tissue compared to the control groupTissue(****p < 0.0001) (Fig. 4).

### 3.6 HIF gene expression is increased in diabetic wound tissues

**Fig. 5:**
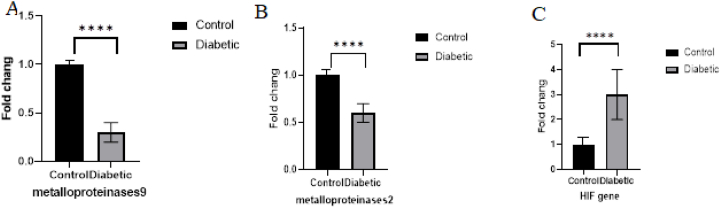
Real-time PCR analysis of genes Metalloproteinase9,Metalloproteinase2 and HIFin Diabete group compared to the control group. Data showed that the transcription level of Metalloproteinase9 and 2 was down-regulated in Diabetic group(****p < 0.0001 (n = 3)). the transcription level of HIF was up-regulated in Diabetic group(****p < 0.0001 (n = 3)).

According to the results, expression of metalloproteinase 2 and 9 genes was showed Tissues with diabetic ulcers showed decreased compared with the controlgroup(****p < 0.0001) (Fig. 5AandB).Data showed that the expression of HIF gene was increased in diabetic ulcer tissue compared to the control (****p < 0.0001) (Fig. 5C).

## 4 Discussion

Redox signaling is a critical regulator in the process of wound healing. This signaling pathway can be effective in the development or healing of diabetic ulcers through the extracellular matrix [17].

In this study,the pathological evaluation results showed an increased amount of collagen and elastin fibers as well as Atrophy lipid levels in the tissues isolated from amputated limbs in patients with diabetic ulcers compared to non-ischemic tissues from patients with limb ischemia as the control group. The MDA content was assessed for lipid oxidation evaluation and was significantly higher in diabetic ulcers than the control group, while the total thiol content, an indicator of protein oxidation, was significantly lower in the diabetic patients than the control group. The expression levels of genes metalloproteinase 2 and metalloproteinase 9 were decreased in the diabetic ulcerous tissues compared to the tissues from the control group, while the HIF expression level was increased in the study group compared to the control group.

The role of MMP in the chronic wound development is mediated by delayed inflammation and the production of high levels of proteinases that degrade the essential components for wound healing, growth factors, various receptors, and ECM proteins( [18,19]). This leads to an increase in MMP and inflammatory cytokines and a reduction of growth factors. When the wound healing begins due to various treatments, an increased mitogenic activity, decreased protease activity, and decreased inflammatory cytokine levels are observed [20]. MMPs play a key role in the development and healing of diabetic ulcers [21]. Chronic diabetic ulcer development is due to delayed inflammation and the production of high levels of proteases, including metalloproteinases( [22,23]). However, increased mitogenic activity and decreased protease activity occurs with therapeutic measures, diabetes improvement, and wound healing [24]. A significantly decreased level of two proteases, metalloproteinase 2 and metalloproteinase 9, was observed in the present study. This can be due to long-term diabetes treatments and chronic ulcers that have led to amputation due to a lack of favorable outcomes of medical treatments. Unlike proteases, angiogenic factors such as HIF can be effective in the process of repair and revascularization in damaged diabetic tissues [25]. HIF production can be induced by various factors, the most important of which is tissue hypoxia [26]. Lower extremity ischemia can occur in some cases of diabetic ulcers. Perfusion is impaired due to ischemia, which will lead to hypoxia and HIF expression stimulation.

Panpan et al. showed that hypoxic conditions have inhibitory effects on metalloproteinase 9 expression through miR-126 production [27]. In this regard, the study by Rahat et al. had similar results on the effect of hypoxia on the reduction of metalloproteinase 9 [28]. Also, the findings of the study by Ying et al. showed a decreased level of metalloproteinase 2 and metalloproteinase 9 under hypoxic conditions, both in vivo and in vitro [29]. On the other hand, the HIF factor itself can lead to ECM deposition in the tissue [30]. ECM deposition subsequently increases the tissue pressure, and the collagen I-to-collagen III ratio increases in collagen accumulation due to hypoxia( [31,32]). This eventually leads to tissue hardness that subsequently decreases tissue flexibility, especially vascular flexibility, leading to tissue ischemia( [33,34]).

Apart from hypoxia, ROS substances can also be effective in higher expression of HIF [35]. In diabetes, the ROS levels can be increased due to high blood glucose [36]. In this study, the MDA level, which was used to assess lipid oxidation, was higher in the diabetic group than the control group, while the total thiol was lower than diabetic group compared to the control group. Another study by Esteghamati et al. on the oxidation level in type 2 diabetic patients showed that the MDA level was increased in type 2 diabetic patients while the thiol content did not show any significant difference between the two groups [37]. In the present study, Atrophy lipid levels were observed in pathological evaluations, while the thickness of protein fibers was higher in the diabetic group than the normal tissue. Also, the NO levels were higher in the diabetic group than in the control group. NO plays an important role in the process of wound healing. NO can play a role in wound healing by increasing angiogenesis and matrix deposition [38]. Also, NO can induce angiogenic factors such as HIF. On the other hand, it can increase the thickness of extracellular matrix fibers such as collagen. Povolny et al. showed that NO treatment was effective in patients with diabetic foot ulcers [39]. Also, the study by Eberhardt et al. showed that NO could indirectly decrease metalloproteinase 9 [40].

## 5 Conclusion

The diabetic foot ulcers leading to limb amputation are not necessarily due to ECM destruction and alteration. In most patients, these ulcers occur in the lower extremities, which are exposed to weight-induced pressure and external stimuli. These are also prone to ischemia and hypoxia. Most malignant wounds leading to amputation are also affected by hypoxia and malperfusion. However, at first, the HIF secretion due to hypoxic conditions is beneficial for matrix deposition and prevents protease activity, but if the hypoxia persists, it can eventually lead to tissue failure and amputation.

## Author statement

Conceptualization: Dr.shirin Saberianpour. Methodology: Dr.shirin Saberianpour,Dr sakineh amouian,Dr Rahimi. Software: Dr.shirin Saberianpour. Validation: Dr Modaghegh. Formal analysis: Dr.saberianpour. Investigation: Dr.saberianpour. Resources: Dr Modaghegh,Dr,Jalili shahri. Writing - Original Draft; Dr.saberianpour. Writing - Review & Editing; Dr.saberianpour,Dr.Jalili shahri. Visualization; Dr.saberianpour. Supervision; Dr.Modaghegh,Dr.saberianpour. Project administration; Dr.Modaghegh. Funding acquisition; Dr saberianpour.

## Ethical code

IR.MUMS.MEDICAL.REC.1399.439.

## Declaration of Competing interest

There is no conflict of interest.
